# Involvement of p38 MAPK in Synaptic Function and Dysfunction

**DOI:** 10.3390/ijms21165624

**Published:** 2020-08-06

**Authors:** Chiara Falcicchia, Francesca Tozzi, Ottavio Arancio, Daniel Martin Watterson, Nicola Origlia

**Affiliations:** 1 Institute of Neuroscience, Italian National Research Council, 56124 Pisa, Italy; chiara.falcicchia@in.cnr.it; 2 Bio@SNS laboratory, Scuola Normale Superiore, 56124 Pisa, Italy; francesca.tozzi@sns.it; 3 Taub Institute for Research on Alzheimer’s Disease and the Aging Brain, Columbia University, New York, NY 10032, USA; oa1@cumc.columbia.edu; 4 Department of Pharmacology, Northwestern University, Chicago, IL 60611, USA; d.m.watterson@gmail.com

**Keywords:** p38-MAPK α inhibitor, Alzheimer’s disease, synaptic plasticity, neuroinflammation, β-amyloid, Tau

## Abstract

Many studies have revealed a central role of p38 MAPK in neuronal plasticity and the regulation of long-term changes in synaptic efficacy, such as long-term potentiation (LTP) and long-term depression (LTD). However, p38 MAPK is classically known as a responsive element to stress stimuli, including neuroinflammation. Specific to the pathophysiology of Alzheimer’s disease (AD), several studies have shown that the p38 MAPK cascade is activated either in response to the Aβ peptide or in the presence of tauopathies. Here, we describe the role of p38 MAPK in the regulation of synaptic plasticity and its implication in an animal model of neurodegeneration. In particular, recent evidence suggests the p38 MAPK α isoform as a potential neurotherapeutic target, and specific inhibitors have been developed and have proven to be effective in ameliorating synaptic and memory deficits in AD mouse models.

## 1. P38 Mitogen-Activated Protein Kinases (p38-MAPK)

The mitogen activated protein kinases (MAPKs) are serine and threonine protein kinases expressed in neuronal and non-neuronal cells in a mature central nervous system (CNS) during a dynamic state in response to various external stimuli, such as growth factors, glutamate and hormones, cellular stress, and pathogens [1]; they mediate proliferation, differentiation, and cell survival [2]. Depending on the context in which MAPKs are activated, they perform specific biological functions that can be therapeutically exploited. The basic module of MAPK cascades consists of three kinases that act in a sequential manner, namely, MAP kinase kinase kinase (MAPKKK) → MAP kinase kinase (MAPKK) → MAP kinase (MAPK) [3,4]. There are more than a dozen MAPK enzymes, but the best known are the extracellular signal-regulated kinases 1 and 2 (ERK1/2), ERK5, c-Jun amino-terminal kinases 1 to 3 (JNK1 to −3), and p38 (α, β, γ, and δ) families [5]. The latter two are also known as the stress-related protein kinases, because they are strongly activated in several pathologic processes, including β-amyloid neurodegeneration associated with Alzheimer’s disease [6,7,8,9]. In particular, mammalian cells are known to express four different genes encoding p38 MAPK isoforms (p38α, p38β, p38γ, and p38δ), which retain a high sequence homology between each other; p38α is 75% identical to p38β and shares 62% and 61% identical protein sequences with p38γ and p38δ, respectively. In addition, p38γ shares around 70% identical sequence with the p38δ isoform. Among them, p38α and p38β are ubiquitously expressed and are mainly involved in inflammatory disorders, whereas p38γ and p38δ are expressed in a tissue-specific manner [10]. They all differ in their expression patterns, substrate specificities, and sensitivities to chemical inhibitors [11]. Each isoform of the p38 MAPK enzyme is activated by dual phosphorylation of the threonine and tyrosine residues. Dual phosphorylation, by either MAP kinase kinase 3 (MKK3) or MAP kinase kinase 6 (MKK6), induces global conformational reorganizations that allow for the binding of ATP and the desired substrate [2]. Many p38 MAPK targets have been described, including protein kinases (MAPK-activated protein kinases, MAPK- interacting kinase, and mitogen- and stress-activated kinase), which in turn phosphorylate transcription factors (p53, ATF-2, NFAT, and STAT1), cytoskeletal proteins (e.g., the microtubule-associated protein Tau), and other proteins with enzymatic activity, such as the glycogen synthase and cytosolic phospholipase A2 [1]. The lack of specific inhibitors for p38γ and p38δ have made the elucidation of the biological roles played by these two p38 isoforms compared to p38α and p38β more difficult. However, the use of knockout mouse models has allowed for demonstrating, for example, that p38γ can bind to the PDZ domain of a variety of proteins, such as PSD95, and modulate their phosphorylation state [12,13,14], while p38δ can phosphorylate Tau and seems to play a role in cytoskeletal remodeling [15]. Immunohistochemistry techniques have been used to study the localization of the main p38 MAPK isoforms in adult mice brains, which demonstrated the presence of p38α and p38β in different regions, including the cerebral cortex and the hippocampus [16]. Their different distribution among cell types was further characterized, showing a predominant neuronal expression for p38α, while p38β is also highly expressed in glial cells [16]. Regarding their subcellular localization in CA1 hippocampal neurons, p38α was found to be widely distributed in the different neuronal compartments, including dendrites, cytoplasm, and nucleus, while p38β was mostly localized at a nuclear level [2,17]. p38α plays a critical role in cellular response to infection related stressors (e.g., lipopolysaccharide (LPS)) [18] and became a drug development target in order to block cytokines production [19]. Moreover, the identification of roles independent of infections led to the extension of what has been called “sterile inflammation” (e.g., injury, illness, or aging). In particular, the activity of p38α has been associated with (a) the progression of the expression of protein markers of the aging phenotype [20,21,22]; (b) the development of inflammation and oxidative stress [10,23] associated with neurodegeneration, including Alzheimer’s [24,25,26], lipopolysaccharide (LPS) [27,28], and Parkinson’s [29,30] diseases; cardiovascular [31] and musculoskeletal diseases; diabetes [32]; rheumatoid arthritis [33]; and toxin-induced preterm birth [34]. Importantly, small molecule inhibitors of the p38 MAPK family have been developed, and show efficacy in blocking the production of proinflammatory cytokines, such as interleukin (IL)-1β and tumor necrosis factor alpha (TNF-α) [35]. Moreover, translational studies identified p38 α as one of several pathophysiology biomarkers in acute brain injury, progressive neurodegenerative disease, psychiatric disorders, and therapy induced drug-resistance [36]. In the present review, we provide an overview of the involvement of p38 MAPK in the regulation of synaptic plasticity, its implication in an animal model of neurodegeneration, and its potential as a neurotherapeutic target.

## 2. p38 MAPK and Synaptic Function

**Figure 1:**
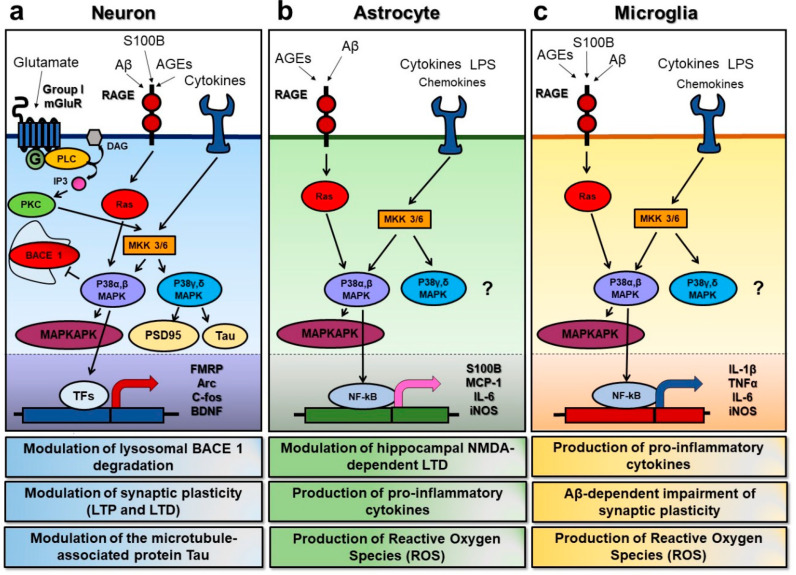
Overview of the p38 mitogen activated protein kinase (MAPK) signal transduction pathways in neurons, astrocytes, and microglia. p38 MAPK can be activated in response to various extracellular stimuli, such as glutamate, advanced glycation endproducts (AGEs), cytokines, and chemokines, leading to cell type-specific downstream effects. (**a**) In neuronal cells, the activation of the metabotropic glutamate receptors of group I (group I mGluRs) can turn on phospholipase C (PLC) and promote the phosphorylation of MAP kinase kinase 3/6 (MKK 3/6), with the subsequent activation of the p38 MAPKs. The α and β isoforms of p38 MAPK have been shown to inhibit beta-secretase 1 degradation, to promote the activation of MAP kinase activated protein kinase (MAPKAKP), and to act on specific transcription factors (TFs) to induce changes in the expression of the key proteins involved in synaptic plasticity, such as the fragile X mental retardation protein (FMRP), the activity-regulated cytoskeleton-associated protein (Arc), the c-fos protein, and the brain-derived neurotrophic factor (BDNF). On the other hand, the γ and δ isoforms seem to have a role in the modulation of synaptic proteins, such as postsynaptic density protein 95 (PSD95) and the microtubule-associated protein Tau. Furthermore, AGEs binding to the receptor for advanced glycation endproducts (RAGE) can turn on the Ras protein, and predominantly lead to the activation of p38 α and β isoforms, while cytokines and chemokines can trigger p38 MAPK by acting through their specific receptors and via MKKs. (**b**) In astrocytes, AGEs and pro-inflammatory molecules such as lipopolysaccharide (LPS) can lead to p38 MAPK activation as well. Moreover, p38 activation in this specific cell type has been demonstrated to modulate hippocampal n-methyl-d-aspartate (NMDA)-dependent long-term depression (LTD), to induce the production of the S100 calcium binding protein (S100B), of pro-inflammatory molecules such as the monocyte chemoattractant proten-1 (MCP-1) and interleukin-6, and to increase the production of reactive oxygen species (ROS) via the expression of inducible nitric oxide synthase (iNOS). (**c**) In microglia, the signaling pathways upstream of p38 MAPK activation are very similar to those found in astrocytes. However, in addition, they lead to the production of pro-inflammatory cytokines such as interleukin-1β, the tumor necrosis factor α, and IL-6, and to increase ROS production, microglial activation of the p38 α isoform has been demonstrated to play a key role in the Aβ-dependent synaptic dysfunction.

Several proteins have been found to be phosphorylated by MAPK, which implicates the role of this enzyme in a large range of cellular functions [5]. p38 MAPK is highly expressed in brain regions that are crucial for learning and memory, and is now emerging as a key player in the synaptic regulation and function. In recent decades, many reports have shown that the p38 MAPK signaling pathway plays important roles in synaptic plasticity (Figure 1).

For example, at the level of hippocampal formation, which is known to express long-term forms of synaptic plasticity, such as long-term potentiation (LTP) and long-term depression (LTD) [11], more information has been collected about the molecular pathways underlying these opposing forms of synaptic modifications. The mechanisms involved depend on the specific synapse and circuit, and the different type of stimulation pattern used to induce LTP and LTD [37]. p38 MAPK has been mostly implicated in synaptic depression, either the n-methyl-d-aspartate (NMDA)R-dependent or the mGluR-dependent form. The activation of NMDAR or metabotropic glutamate receptors (mGluRs) [11] triggers a diversity of signaling cascades, which results in a rapid and sustained decrease in synaptically evoked excitatory postsynaptic potentials (EPSPs). The activation of p38 MAP kinase in the hippocampus has been found to be necessary for the induction of mGluR-dependent LTD at the excitatory synapses between the CA3 and CA1 pyramidal neurons [38]. Indeed, p38 MAPK has been found in the hippocampus and has been demonstrated to be activated in response to a synaptic stimulation protocol that induces LTD [38]. In addition, it has been shown that a specific genetic ablation of the p38α isoform virtually abolishes NMDA-dependent LTD when targeting astrocytes, while producing no effect or slightly enhancing LTD when targeting neurons. These data indicate that astrocytic p38α is involved in activity-dependent glutamate release from astrocytes, contributing to astrocyte-to-neuron communication [39]. The authors concluded that the activity of p38α MAPK in the astrocyte contributes to hippocampal NMDA-dependent LTD, and is capable of modulating long-term memory in vivo [39]. Another study investigated the changes in the mRNA expression levels of p38 MAPK, demonstrating its implication in the induction of LTD in response to low-frequency stimulation (LFS) [40]. The requirement of p38 MAPK for the expression of NMDA-dependent LTD was also suggested in other brain circuits using entorhinal cortex slices, where the application of the SB203580 MAPK inhibitor completely suppressed LFS-induced LTD in superficial layer II [41]. Furthermore, in the mouse primary visual cortex, p38 MAPK has been demonstrated to mediate anisomycin-induced LTD by promoting α-amino-3-hydroxy-5-methyl-4-isoxazolepropionic acid (AMPA endocytosis). In fact, anisomycin administration produced a time-dependent decline in field excitatory post-synaptic potentials (fEPSPs) amplitude in acute brain slices of V1, and this decline could be rescued by the application of SB203580 [42]. However, SB203580 is proposed as a multi-target kinase inhibitor, which makes its use in support of a specific p38MAPK biological role more questionable [43].

Although, as reported above, p38 MAPK inhibition at the CA3-CA1 synapses does not affect LTP induction, p38 MAPK still appears to have a role in long-term potentiation, at least in pathological models. For example, reducing p38 MAPK activation by improved synaptic plasticity in angiotensin II-dependent hypertensive mice, either through genetic knock-down or pharmacological inhibition with SKF86002, as assessed by the LTP recording in the hippocampal slices [44]. Similar results were obtained in the entorhinal cortex (EC), where [45,46] the suppression of synaptic plasticity by the administration of Amyloid-β in slices could be prevented by a selective pharmacological inhibition of p38 MAPK using the MW 108 compound. In a more recent study, it was found that inhibition of the MAPK signaling pathway in an AD mouse model resulted in an improvement in hippocampal LTP [47]. Indeed, these data demonstrate that the impairment of LTP observed in APP/PS1 mice, was reversed by up-regulating mitogen-activated protein kinase (MAPK) phosphatase 1 (MKP-1), an essential negative regulator of MAPKs [47]. Finally, the importance of environmental factors in determining the role of p38 MAPK signaling cascade in LTP induction has been demonstrated [37]. In particular, the p38 MAPK was not required for hippocampal LTP in adolescent mice reared in standard conditions, but its activation was involved in LTP expression after exposure to an “enriched environment” [37]. Moreover, the NMDA glutamate receptor-dependent activation of p38 MAPK rescued the LTP in adolescent Ras-GRF knockout mice. This study revealed a new level of cell signaling control, whereby environmental factors influence the efficacy of a specific cascade to control LTP expression in adolescent animals [37].

## 3. p38 MAPK Neuroinflammation and Synaptic Dysfunction

The process of acute inflammation in mammalian tissue is one of extreme importance, as it is the immediate cellular response to injury and it is a defensive mechanism to prevent damage to the cells. The p38 module plays a critical role in normal immune and inflammatory responses; indeed, many studies have revealed its involvement in the production of inflammatory cytokines leading to chronic inflammation [11]. Thus, p38 is activated by numerous extracellular mediators of inflammation, including cytokines, chemoattractants, chemokines, and bacterial lipopolysaccharide (LPS). However, a major function of p38 isoforms is in turn the production of proinflammatory cytokines, and it has been proven that p38 can regulate cytokine expression by modulating transcription factors, such as nuclear factor-kB (NF-KB) [48], or at the mRNA level, by modulating their stability and translation through the regulation of MNK1 [49] and MNK2/3 [50]. It is also known that chronic inflammation occurs when there are persistent inflammatory stimuli that can have a damaging rather than protective effect. For example, chronic glial cell activation is present in neurodegenerative diseases [2]. The p38 MAPK pathway contributes to neuroinflammation mediated by glial cells, including microglia and astrocyte, and p38 α appears to be the main isoform involved in the inflammatory response [5]. In the brain, one of the physiological roles of microglia and astrocytes is to respond to stress and other cellular stimuli, defend the brain tissue, and take part in an inflammatory response by acting as mediators in inflammation and neuroprotection. Changes in morphology and transcriptional activation take place in the transition of microglial cells from a resting state, which exhibit a ramified morphology at the microscope, to an activated state with less extensive branching and processes. Activated microglia and reactive astrocytes are able to produce reactive oxygen species (ROS) and neurotoxic molecules that can induce molecular processes leading to neuronal death [51], but a prolonged and sustained activation of glial cells can result in an exaggerated inflammatory response and, as a result, cause neuronal cell death through the elevated release of proinflammatory cytokines, which have a potential neurotoxic effect, leading to increased neurodegeneration [2]. In addition to cellular damage, high concentrations of pro-inflammatory cytokines have been shown to affect neuronal synaptic functioning via p38 MAPK signaling in a variety of brain regions. For example, p38 MAPK mediates the inhibitory effect of the pro-inflammatory cytokine interleukin-1β (IL-1β) against LTP in the rodent dentate gyrus [52]. In the CNS, IL-1β levels increase in response to a number of different stimuli, such as the peripheral administration of lipopolysaccharide (LPS) [53], traumatic brain injury [54], acute stress [55], and β-adrenoceptor agonist administration [56]. IL-1β has been demonstrated to inhibit both NMDA- dependent and -independent forms of LTP in the hippocampus, and to progressively increase during aging in parallel with the age-related impairment of LTP in rodents [57], suggesting that it may represent one possible cause of cognitive decline. Indeed, two weeks of IL-1β overexpression in an inducible transgenic mouse was demonstrated to impair long-term contextual and spatial memory, but did not affect short-term and non-hippocampal memory [58]. Moreover, besides IL-1β, other pro-inflammatory cytokines have been shown to influence synaptic plasticity via p38 MAPK signaling, such as tumor necrosis factor alpha (TNF-α). TNF-α inhibits LTP in CA1 and DG in the rat hippocampus at pathophysiological levels [59], and the inhibition of p38 MAPK reverses the effect of TNF-α on early phase LTP without affecting late LTP [60]. Therefore, p38 MAPK appears to play an important role in cytokine-induced synaptic dysfunction, and there is evidence that it is also a key molecule in neurodegenerative diseases. Neurodegeneration represents a common pathological condition to several brain disorders, including Alzheimer’s disease (AD), multiple sclerosis (MS), Parkinson’s disease (PD), Huntington’s disease (HD), and amyotrophic lateral sclerosis (ALS), and highlighting the functional role of specific p38 MAPK substrates will be of particular importance, as these could be potential signaling targets.

### p38 MAPK, AD Neurodegeneration and Synaptic Dysfunction

Alzheimer’s disease (AD) is the most prevalent age-related, progressive, and irreversible neurodegenerative disorder, characterized by memory dysfunction and cognitive impairment that are thought to result from the formation in the brain both of senile plaques containing amyloid-β (Aβ), as well as neurofibrillary tangles containing the microtubule-associated protein tau [36]. Aβ toxicity and tau hyperphosphorylation increase the activation of mitogen-activated protein kinase (MAPK) and MAPK signaling [61]. p38 MAPK is one of the key regulators of Aβ induced toxicity from this family [62]. In this regard, the Aβ-induced synaptic dysfunction has been well characterized. First, it has been demonstrated that either a synthetic form of Aβ 1-42, in the nanomolar range, or cell-derived naturally secreted Aβ oligomers, have a strong inhibitory effect on the induction of hippocampal LTP, both in vitro and in vivo in the CA1 area [63,64]. It has been shown that higher Aβ levels are also able to depress glutamatergic synaptic transmission and surface receptor number [65]. This effect was described as a partial occlusion of LTD, and suggests that Aβ-induced depression shares some mechanisms also necessary for LTD expression, including activation of the p38 MAPK pathway. However, it was first demonstrated that the activation of p38 MAPK is involved in the inhibition of hippocampal LTP by Aβ [63]. In subsequent studies, the differential activation of stress related kinases, p38mapk, and JNK, involved in the progressive Aβ-dependent synaptic dysfunction, has been investigated in entorhinal cortex slices. A concentration-dependent effect of Aβ was described, with a lower nM concentration that selectively impairs LTP through the neuronal activation of p38 MAPK [45], while increasing Aβ concentration up to 1 μM induces specific phosphorylation of both p38 MAPK and JNK that would consequently affect glutamatergic synaptic transmission and the expression of LTD [41]. In particular, it was reported that Aβ was able to phosphorylate p38MAPK in cultured cortical neurons at concentrations and incubation times comparable with those used for LTP. Moreover, a dual role emerged for p38 MAPK, as it was required for LTD expression, but also contributed to LTD impairment induced by higher Aβ levels. Notably, Aβ exposure increased the phosphorylated levels of p38 MAPK, which were further enhanced after low frequency stimulation (LFS), the protocol used to induce LTD and capable of phosphorylating p38 MAPK [41]. Concerning the possible cell surface targets that are able to bind Aβ and trigger p38 MAPK cascade, the receptor for advanced glycation end-products (RAGE) was identified as being capable of binding Aβ, in monomeric, fibrillized, and oligomeric forms, and to contribute to the progressive deleterious effects of the Aβ (1-42) peptide on EC synaptic function. In particular, Aβ-mediated the enhancement of p38 MAPK phosphorylation in cortical neurons, and was reduced by blocking antibodies to RAGE [45]. Moreover, increased phospho-p38 MAPK after Aβ exposure was reduced in EC slices from RAGE defective mice [41].

Indeed, the activation of p38 MAPK has been verified in the brain during the early stages of AD, both clinically [6,66] and in mouse models [11,62,67,68].

Different transgenic mouse models have been used to investigate the role of p38 MAPK. The link between the Aβ/RAGE axis and p38 MAPK over-activation has been confirmed in double transgenic mice (APPswe/Ind J20 expressing defective-RAGE in microglia), demonstrating that the activation of RAGE inflammatory signaling in vivo caused by an Aβ enriched-environment represents an important early event during progressive EC dysfunction. In the APPJ20 model, neuronal plasticity is progressively impaired in EC slices, while the inhibition of RAGE signaling in microglia ameliorates synaptic and behavioral impairment in APPJ20xDNMSR mice, reducing the neuronal activation of p38MAPK. This finding supports the hypothesis that microglial RAGE interaction with Aβ may therefore contribute to triggering p38 MAPK signaling involved in cognitive dysfunction in vulnerable brain areas, resulting in the spreading of AD pathology.

More recently, double transgenic mice, which express both the human APP mutation and endophilin A1(EP), demonstrated that the upregulation of the EP expression in Aβ-rich environments leads to changes in both hippocampal LTP and learning and memory. Specifically, EP, a synaptic protein elevated in AD patients and AD transgenic animal models, increases cerebral Aβ accumulation. The EP-mediated signal transduction involved reactive oxygen species (ROS) and p38 MAPK, contributing to Aβ-induced mitochondrial dysfunction, synaptic injury, and cognitive decline. The neurodegenerative phenotype could be rescued by blocking either the ROS or p38 MAP kinase activity [69,70].

Evidence also exists that genetically targeting the alpha-isoform of p38MAPK is sufficient to ameliorate synaptic dysfunction. With immunological and biochemical methods, it has been observed that the reduction of the p38α MAPK expression facilitates the lysosomal degradation of BACE1, a key enzyme in Aβ generation that is potentially up regulated by neuroinflammation. This led to an attenuation of Aβ protein generation in the brain of APP/PS1 double transgenic mice, suggesting that p38α MAPK plays a role in the process of Aβ deposition in vivo [71]. Moreover, the selective pharmacological inhibition of p38α MAPK was neuroprotective in either amyloid or tau models of AD [46,68]. However, the role of p38α in neurodegeration can be different depending on the model. A recent report demonstrated that neuron-specific p38α-knockout mice show increased levels of anxiety in behaviour tests, an effect that was mediated by increased JNK activity [72]. 

The novel isoform selective p38α MAPK inhibitor was tested in two different animal models characterized by early synaptic and behavioral dysfunctions, and was found to be effective in ameliorating hippocampal-dependent associative and spatial memory. The first evidence was that selective p38α MAPK inhibition was capable of reducing LTP and memory deficits in the APP/PS1 Tg model, whose main feature is represented by Aβ deposition [25]. In contrast, no overexpression of APP was present in the APP/PS KI mouse, which can be considered a model of aging with physiological levels of APP. This mouse shows a slower pathology progression and offers the opportunity to study either the early or the late stage of neurodegeneration. In 11-month-old KI mice, the overproduction of cytokine leads to a spatial memory deficit that can be reduced by pharmacological treatment targeting p38α MAPK [25]. In addition, several inhibitors of p38α MAPK were effective in ameliorating the behavioral deficit in a mouse model of tau-related neurodegeneration, the reversible transgenic (rTg4510) mouse model. These mice overexpressed a human, mutant tau form (P301L) and developed age-related cognitive impairment, neurofibrillary tangles, and neuronal loss [73]. Overall, this evidence favors the hypothesis that p38 MAPK and, in particular, the α isoform, contribute to different pathological process, and therefore represents a promising therapeutic target for the treatment of AD [11,44]. 

An opposite role in neurodegeneration emerged for p38γ MAPK. This isoform mediates phosphorylation on tau at Threonine-205 (T205), a site-specific phosphorylation that improved memory deficits in APP transgenic mice [43], in particular interfering with the synaptic action of Aβ on glutamatergic neurotransmission [74,75].

## 4. Small Molecules Targeting p38α MAPK

Serine–threonine (S/T) protein kinases are important for CNS function and have been implicated in pathophysiology, yet there is a dearth of highly selective, CNS-active kinase inhibitors for in vivo investigations.

**Figure 2:**
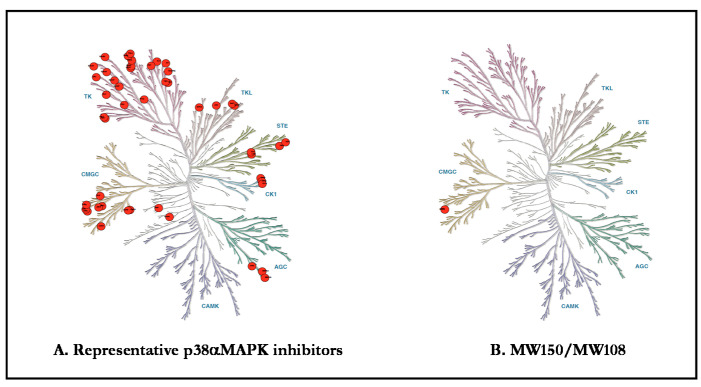
Kinome target selectivity of p38 MAPK Inhibitors. Differences in off-target kinase and GPCR liabilities provide potential explanations for pharmacological differences. Red circles denote kinase inhibition below the canonical IC50 < 1 µM. (**A**) Common off-target kinases among widely used p38α MAPK inhibitors: VX-745 (neflamapimod) includes ABL1, ABL2, p38β, PDGFRβ, and SRC; BIRB-796 includes BLK, CDK5, CDK8, DDR1, DDR2, EPHA3, EPHA7, EPHA8, EPHB2, p38β p38γ, FLT1, FRK, NTRK1, JNK1, JNK2, JNK3, KIT, MAP4K4, MRCKβ, PTK2β, RET, SLK, STK10, TIE1, TIE2, TNIK, TRKB, TRKC, and ZAK; and SB203580 includes BRAF, CIT, CK1δ, CK1ε, DMPK, GAK, JNK2, JNK3, NLK, p38β, RIPK2, STK36, and TNIK. (**B**) MW150 and MW108 have IC50 <1 µM for p38α MAPK in kinome-wide hierarchal screens.

A challenge for all of life sciences is that approved protein kinase inhibitor drugs and research molecular probe inhibitors are multi-kinase inhibitors. Results should be taken with caution when using small-molecule inhibitors of protein kinases to investigate the physiological roles of these enzymes [76,77,78,79,80,81]. Figure 2 presents a graphical presentation of the kinome off-target issue for the S/T protein kinase, p38α MAPK, which is the focus of this review. As a consequence, the outcome of experiments are confounded by the use of p38MAPK inhibitors that also inhibit other kinases such as ABL1, ABL2, p38β, p38γ, CK1δ, CK1ε, PDGFRβ, SRC, BLK, CDK5, CDK8, DDR1, DDR2, EPHA3, EPHA7, EPHA8, EPHB2, FLT1, FRK, NTRK1, JNK1, JNK2, JNK3, KIT, MAP4K4, MRCKβ, PTK2β, RET, SLK, STK10, TIE1, TIE2, TNIK, TRKB, TRKC, ZAK, BRAF, CIT, DMPK, GAK, JNK2, JNK3, NLK, RIPK2, STK36, or TNIK. For example, MW150 has no direct effect on amyloid plaques [25,82] and avoids ABL as a target [25]. The pharmacological difference from p38MAPK inhibitors that have off-target ABL inhibition might be explained by the known effects of ABL inhibitors (e.g., Imatinib, Nilotinib and Bosutinib) on amyloid plaques [83,84,85]. Similarly, the medicinal chemistry refinement of the original fragment expansion hit, MW069a, to yield the more selective MW181 and MW108 class of refined inhibitors involved removal of the previously identified liability of protein kinase CK1d inhibition and removal of cross-over to potential excitotoxic GPCR targets. There was a coincident improvement in pharmacological safety as the neurotoxicity seen at high doses of MW069a was removed. The coincident removal of both off-target activities and improvement in pharmacological safety does not allow conclusions about exactly what kinase or GPCR target might represent a risk for higher doses of p38MAPK inhibitors. However, clinical findings and associated animal model studies describe susceptibility to migraine and sleep disorders in the presence of reduced CK1d activity suggesting risk in this off-target kinase [86]. An additional challenge for neurosciences is the fact that most protein kinase inhibitors used in research studies, as well as kinase inhibitor approved drugs, lack sufficient CNS exposure to allow adequate molecular target exposure. The blood–brain barrier challenge is not limited to protein kinase inhibitor drugs, however, as it is estimated that >95% of approved drugs lack sufficient brain tissue exposure. The CNS exposure limitation is most often linked to the molecular properties of the small molecule drugs [78]. However, this is a barrier that can be addressed through medicinal chemistry refinement [73,79].

Recent deliverables for p38α MAPK, MW108, and MW150 [25,73,78] avoid the molecular target selectivity and brain exposure challenges that plagued prior works. They provide precedents for CNS S/T protein inhibitors based on their high kinome selectivity, avoidance of high-risk off-target effects, and in vivo efficacy. The selectivity of MW108 and MW150 was demonstrated by large-scale kinome screens, functional GPCR agonist and antagonist analyses, and selected ion-channel and transporter screens. Furthermore, MW150 treatment at efficacious doses does not produce detectable pharmacodynamic effects in knock-in cells that have the endogenous kinase p38α MAPK replaced with p38α MAPK (T106M), an active p38α MAPK that is MW150 resistant. In vitro and in vivo assays demonstrated cellular target engagement, and dose dependent studies in diverse animal models documented their pharmacodynamic and efficacy functions. For example, the MW108/MW150 series ameliorated beta amyloid-induced and tau-induced synaptic and cognitive dysfunction in neurodegeneration [25,46,73,74,75,87], as well as attenuate behavioral symptoms and pathophysiological biomarkers in a genetic susceptibility model of autism spectrum disorders [88]. Clearly, the MW108/MW150 series allows for the pursuit of preclinical and clinical therapeutic hypotheses involving p38α MAPK that were not feasible previously.

The activation of p38α MAPK mediated signaling cascades are implicated in synaptic dysfunction in neurodegenerative disorders through clinical observations and preclinical investigations. Activation in both neurons and glia offers the unusual potential to generate enhanced phenotypic responses through targeting a single kinase in two distinct cell types involved in pathophysiology progression. The pathophysiology mechanism, plus a highly selective and bioavailable p38α MAPK inhibitor drug, could provide a novel form of pleiotropy (one drug, one target, multiple clinical effects), in contrast to the pleiotropic effects of drugs such as steroids, where the same drug engages multiple molecular targets. The high safety potential for MW150 in preclinical toxicology screens and first-in-human clinical trials suggests that such a pleiotropic mechanism could be highly desirable clinically [73]. However, it raises the need for caution if MW150 is used as a molecular probe reagent in basic research experimental design.

In summary, recent progress has directly addressed the scientific challenges in the development and use of p38α MAPK inhibitors as therapeutic candidates, and greatly improved the delivery of small molecule inhibitors as in vivo research tools. Overall, the paradigm shift and new deliverables allow for more robust testing of various hypotheses about S/T protein kinases, such as p38α MAPK in neuropathology progression and its potential for disease modification.

## Abbreviations

MAPK	mitogen activated protein kinases
CNS	central nervous system
LTP	long-term potentiation
LTD	long-term depression
AD	Alzheimer’s disease
ERK	extracellular signal-regulated kinases
EPSPs	synaptically evoked excitatory postsynaptic potentials
JNK	c-Jun amino-terminal kinases
ATP	adenosine triphosphate
NFAT	nuclear factor of activated T cells
ATF	activating transcription factor
STAT1	signal transducer and activator of transcription
LPS	lipopolysaccharide
IL-1β	interleukin-1β
TNFα	tumor necrosis factor
AGEs	advanced glycation endproducts
mGluRs	metabotropic glutamate receptors
TFs	transcription factors
FMRP	fragile X mental retardation protein
Arc	activity-regulated cytoskeleton
BDNF	brain-derived neurotrophic factor
PSD95	postsynaptic density protein 95
RAGE	advanced glycation endproducts receptor
MCP-1	monocyte chemoattractant proten-1
iNOS	inducible nitric oxide synthase
NMDA	n-methyl-d-aspartate
LFS	low-frequency stimulation
AMPA	α-amino-3-hydroxy-5-methyl-4-isoxazolepropionic acid
Aβ	amyloid-β
EC	entorhinal cortex
Tg	transgenic
APP	amyloid precursor protein
PS1	mutant human presenilin 1
EP	endophilin
NF-KB	nuclear factor-kB
ROS	reactive oxygen species
MS	multiple sclerosis
PD	Parkinson’s disease
HD	Huntington’s disease
ALS	amyotrophic lateral sclerosis
KI	knock in
KO	knock out
S/T	serine-threonine
FPCR	G protein-coupled receptor
